# A national survey of dementia diagnosis and care in English memory services

**DOI:** 10.1186/s12877-026-07155-w

**Published:** 2026-02-19

**Authors:** Oliver Kelsey, Harriet Demnitz-King, Charlotte Kenten, Hannah Chapman, Malvika Muralidhar, Ellen Camboe, Elenyd Whitfield, Sedigheh Zabihi, Emma Williams, Annie Jones, Susan Williams, Charles R. Marshall, Jonathan M. Schott, Ruth Dobson, Sube Banerjee, Claudia Cooper

**Affiliations:** 1 Centre for Psychiatry and Mental Health, Wolfson Institute of Population Health, Queen Mary University of London, Yvonne Carter Building 58 turner Street, London, UK; 2 Department of Primary Care and Mental Health, University of Liverpool, Liverpool, UK; 3 Institute of Neurology, University College London, London, UK; 4 Faculty of Medicine and Health Sciences, University of Nottingham, Nottingham, UK

**Keywords:** Memory services, Health policy, Dementia, Mild cognitive impairment (MCI)

## Abstract

**Background:**

In England, National Health Service (NHS) memory services provide most dementia diagnostic and immediate post-diagnostic care. We aimed to co-design and conduct a survey regarding diagnostic and post-diagnostic care, and perceived readiness for new treatments.

**Methods:**

We invited all memory services in England to complete the survey. We compared services by provider type, investigating whether service characteristics (provider type, rurality, region, referral rates, staffing mix, accreditation) were associated with diagnosis rates and psychological therapy provision.

**Results:**

139/188 (73.9%) memory services participated, 130 (93.5%) provided by mental health/community and 9 (6.5%) by acute trusts. We estimated that English memory services receive 192,418 referrals/year, 98.7% to mental health/community trust services. In these services, the median annual referral rate per Full Time Equivalent (FTE) staff was 100.8 (Interquartile range: 56.7-132.8). Of FTE memory service staff, 14.0% (9.0–19.0%) were doctors. Acute trust-based services reported fewer referrals (45.8, 21.1–99.5) and had more doctors (33.0%, 23.0–43.0% FTE). More acute trust services felt ready to prescribe dementia Disease Modifying Treatments (*N* = 8 [88.9%]) than mental health/community services (*N* = 50, [41.7%]), while fewer acute trusts offered post-diagnostic psychological therapy routinely (*N* = 5 [55.6%]) vs. (*N* = 100 [77.5%]) in community services. NHS region (β = 0.70 [95% Confidence interval (CI): 0.08, 1.32]) and rurality (β = 2.14, [95% CI: 1.32, 2.96]) predicted lower diagnostic rates; regions with highest dementia diagnosis rates (67%+) had more memory service staff relative to the local aged 65 + population size.

**Conclusion:**

We identified marked geographical inequalities. People in regions with less resourced memory services and rural areas had less access to timely diagnosis and care.

**Supplementary Information:**

The online version contains supplementary material available at 10.1186/s12877-026-07155-w.

## Background

**Fig. 1:**
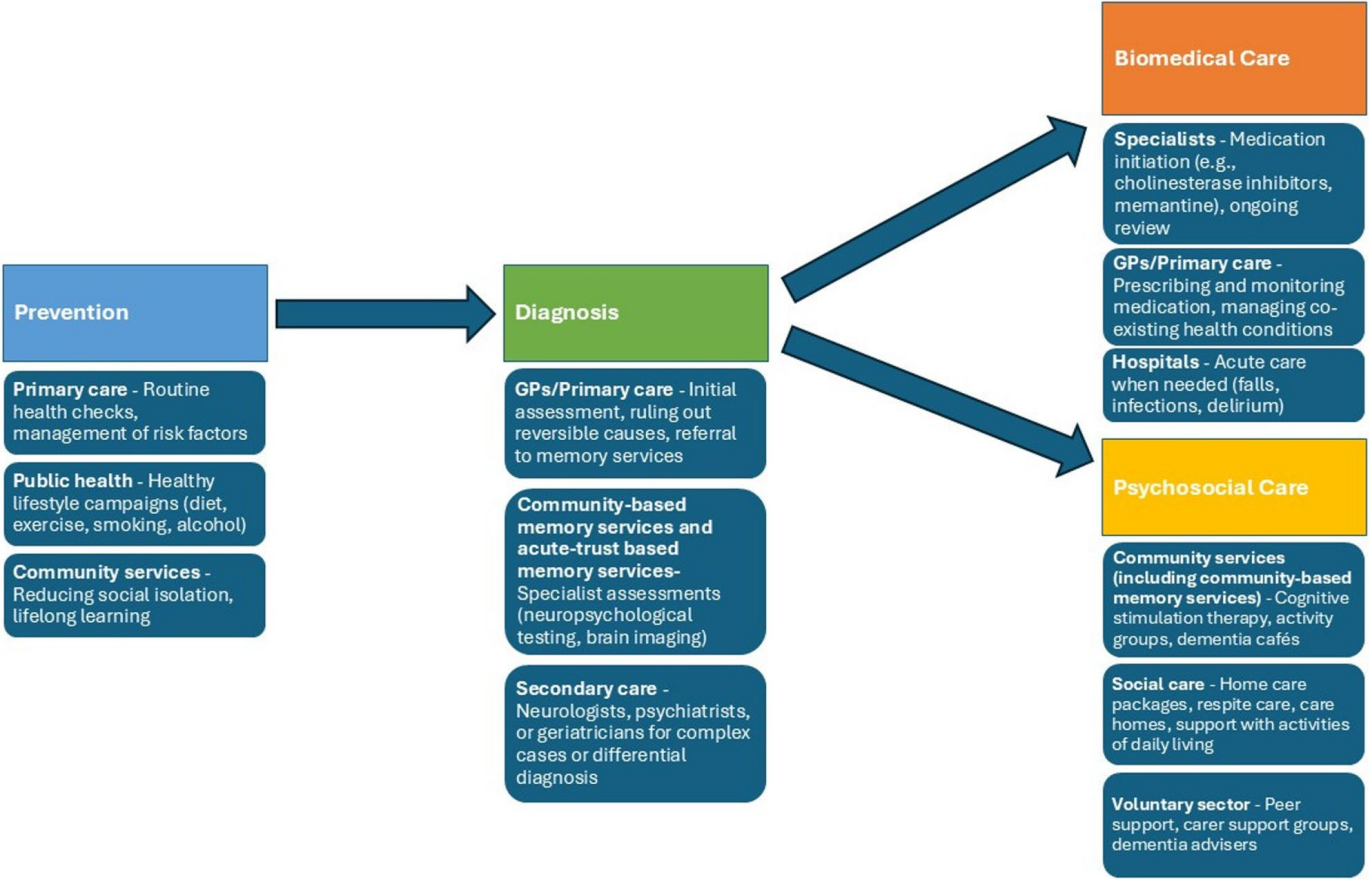
Major dementia service stages in England

An estimated 982,000 people have dementia in the United Kingdom (UK), this is predicted to rise to over 1.4 million by 2040 [1]. In England, most dementia diagnostic assessment and post-diagnostic care is provided by National Health Service (NHS) memory services. Figure 1 depicts the major dementia service stages in England. Most memory services are provided within mental health services, staffed by psychiatrists, while some are run within hospital-based services, primarily by neurologists. The UK National Institute for Health and Care Excellence (NICE) dementia assessment and treatment guideline advocates integrated, multidisciplinary care, diagnostic assessments using validated cognitive tests and structural imaging, and post-diagnostic support, including pharmacological symptomatic treatments where appropriate, and psychosocial and environmental interventions to reduce distress [2].

Memory service audits in England (2019−23) found average waiting times for assessment and diagnosis increased over this time, with longer waiting times in deprived areas. These audits did not include acute NHS Trust based services (henceforth acute trust services). Over two-thirds of audited patients received a diagnosis of “unspecified dementia”, suggesting many people did not access accurate, subtype-based dementia diagnoses. Following diagnosis, only a third of people with dementia eligible for Cognitive Stimulation Therapy (CST) were offered it, with similar proportions of carers offered structured support [3]. A survey of 73 memory services in 2015 found no association between staffing levels and waiting times or service provision [4].

Memory services operate in challenging contexts, as described in the Darzi report on the NHS [5]. Anticipated developments include introducing first generation anti-amyloid Disease Modifying Treatments (DMTs), and blood-biomarker tests which can potentially increase dementia diagnostic accuracy [6]. A 2021 survey found that only 36% of London memory services felt capable of adapting to the demands of delivering DMTs within a year [7]. Implementing these new developments without delivering more inclusive, integrated care will pose challenges [8].

To understand current memory service provision, we completed a comprehensive survey of acute trust and community/mental health trust (henceforth community) based memory services in England. To investigate variations in provision and how they might relate to service delivery, we aimed to:

1. Describe the referral, diagnostic and post-diagnostic procedures of English memory services.
2. Describe the perceived readiness for DMTs and blood biomarkers, comparing acute and community services.
3. Explore whether service characteristics (NHS region, referral rates, staffing mix, provider type and Royal College of Psychiatrists Memory Services National Accreditation Programme (MSNAP) accreditation) are associated with local diagnosis rates and psychological therapy provision.
4. Explore changes in the past five years that staff feel most proud of, those that cause them most concern, and key changes anticipated in the next five years.

## Methods

The cross-sectional survey was conducted by the National Institute for Health and Care Research (NIHR) Dementia and Neurodegeneration Policy Research Unit - at Queen Mary (DeNPRU-QM), commissioned by the English Department of Health and Social Care (DHSC) and approved by National Research Ethics service and Health Research Authority on 14.5.24 (24/IEC08/0008).

### Survey design

We purposively invited a diverse group of people with academic, clinical and lived experience from across English regions and staff roles, and NHS England and MSNAP representatives to three online co-design workshops in March-April 2024. Prior to workshops, we circulated an accessible summary of relevant research [4, 9, 10], audits [3, 11, 12] and policy documents [13, 14]. Using a structure, process and outcome framework, group members proposed and discussed survey items. All attendees were sent the final survey and invited to further comment; a core study group comprising academic, clinical and lived experience agreed the final survey content.

The survey elicited service characteristics including structure (e.g. staffing mix and referral numbers), and processes (how the services diagnosed dementia and provided post-diagnostic services). Respondents were asked about service changes over the last five years, those planned in the next five years and how prepared services were for introduction of blood biomarkers and DMTs. The survey was developed for the purposes of this study and is available in the supplementary materials (S4).

### Survey sample and data collection

We developed a sampling frame of all English memory services from publicly available information; and invited services to participate, between May 2024 and January 2025. Responding services were asked to identify a staff member knowledgeable of service structures and processes, service history and plans. After providing informed consent, the staff member completed the survey with a researcher by video-call, phone or if they preferred directly via the online software survey platform Qualtrics (https://www.qualtrics.com). Participants/services received no compensation.

Researchers used publicly available data to record whether each service was currently MSNAP registered [15] and area-level data regarding:

1. Local Authority District (LAD) rurality, using 2011 Office for National statistics Rural-Urban classifications: 1 (Mainly rural), 2 (Largely Rural), 3 (Urban with significant rural), 4 (Urban with city or Town), 5 (Urban with Minor Conurbation), 6 (Urban with major Conurbation) [16]; we categorised 1 and 2 as ‘rural’ and 3–6 as ‘urban’.
2. Dementia diagnosis rates (the proportion of people aged 65+ with a formal dementia diagnosis, out of all those over 65 estimated to have dementia), obtained from NHS primary care dementia 2024 data [17]. We attributed the relevant LAD diagnostic rate for service catchment areas and calculated the mean diagnostic LAD rates if where catchment areas encompassed >1 LAD (n=25). Where we could not map LADs to catchment areas (n=8), we used rates for service Integrated Care Boards (ICB).
3. The number of older people (aged 65+) living in each region, from publicly available data [18].

### Analysis

Data were exported from Qualtrics and analysed using Statistical Package for the Social Sciences (SPSS; version 29). During data cleaning we removed outlier statistics more than three Standard Deviations (SD) from the mean (*n* = 4). We summarised findings descriptively, partitioning into community and acute services as they operate distinct models. For each region we estimated total overall staff FTE (Full Time Equivalent) of all memory services, imputing the median value for that region for non-participating services. We calculated regional number of older people per memory service FTE and compared this across regions and to regional diagnostic rates.

We used RStudio [19] to map memory service locations. For each service, we calculated the average number of referrals estimated in the past year (referral rate) per staff FTE. We used parametric statistics to describe and analyse data approximating the normal distribution (evaluated graphically) and non-parametric statistics otherwise.

We conducted, linear and logistic regression analyses with: (i) local area diagnostic rates and (ii) any psychological therapies offered as dependent variables. For each outcome, in model 1, we included referral rate: staff ratios; in model 2, we added NHS region and rurality; in model 3 we added: staffing mix (% of FTE staff who were doctors), service type (acute or community) and MSNAP accreditation as independent variables.

We conducted content analysis of responses recorded (primarily by researchers conducting interviews, or by respondents online) to open-ended questions, using published methods [20]. We used the Dementia Well pathway to develop our coding framework, combining deductive and inductive analytic approaches [21]. Two authors (from CC, SZ, OK) independently coded > 10% of responses for each question, developed initial groupings of themes and then met to discuss a coding framework. One author coded the remaining responses.

## Results

### Survey response

Managers and senior staff members from 139/189 (73.9%) English memory services, employed in their current roles for on average 5.2 years (SD = 4.8) completed the survey. Respondents included 52 team managers/leaders, 32 service leads/managers, 19 nurses, nine psychiatrists, four neurologists, two Advanced Clinical Practitioners and two clinical psychologists. Eight respondents, with pan-service operational roles, completed surveys for multiple (< 6) services.

**Fig. 2:**
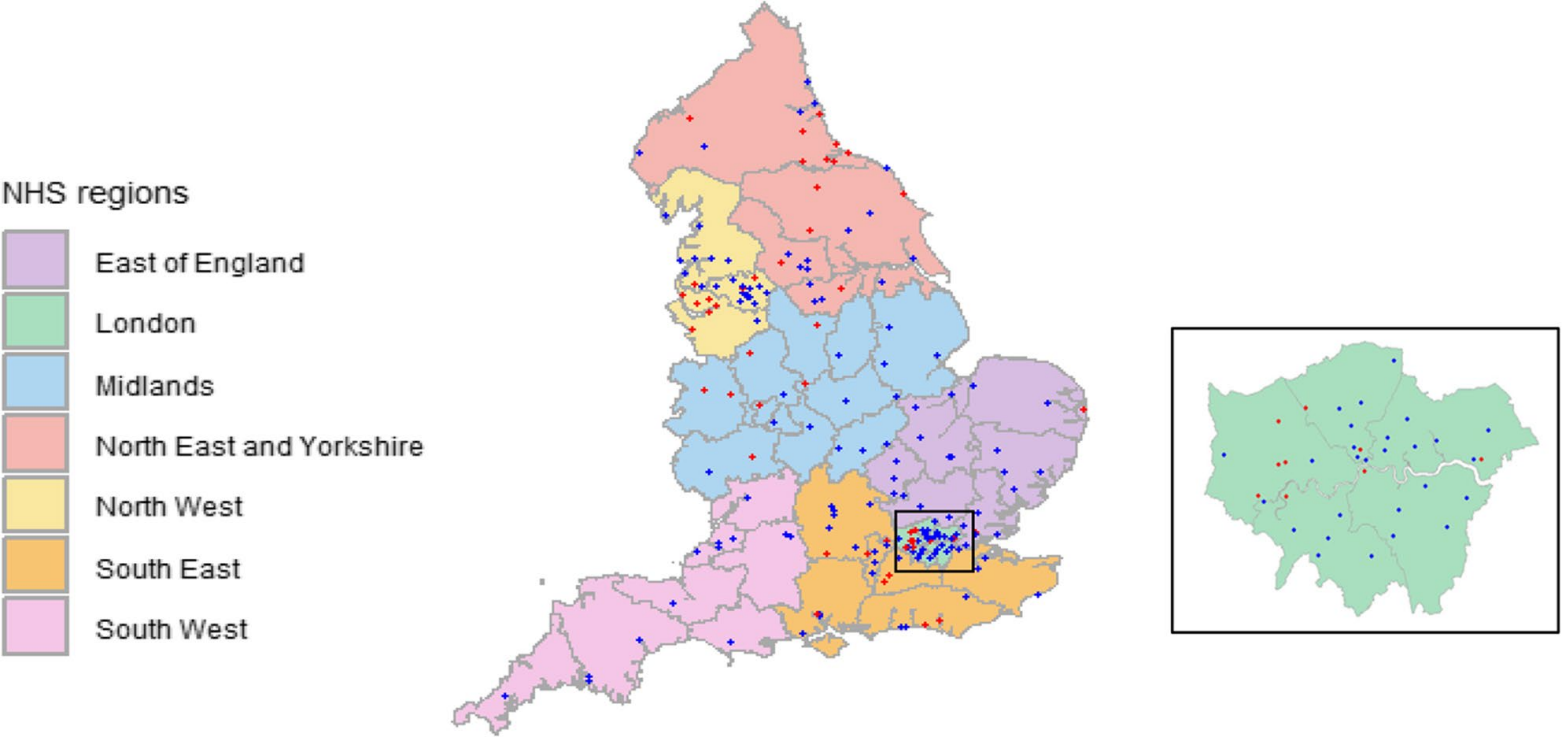
Location of English memory clinics. Blue dots indicate services that participated, red dots services that did not participate; grey boundaries show the 42 English integrated care boards, an inset map shows London services

Figure 2 shows the geographical distribution of participating services. Completion rates were highest in the South West NHS region (12/12 [100.0%] services) and lowest in the North East and Yorkshire (19/33 [57.6%] services). The 49 services not responding were all community services.

132/139 (95%) services were dedicated to memory assessment, while 7 (5%) fulfilled other functions, most commonly also acting as older person’s Community Mental Health Team’s (CMHTs) (5, 71.4%). 47/139 (33.8%) services were MSNAP accredited: 2/9 (22.2%) acute and 45/130 (34.6%) community services.

### Service structure

130/139 (93.5%) participating services were NHS community services (121/130 (93%) provided by mental health trusts, 7 (5.5%) by mental health trusts with General practitioner (GP)/community service providers, and 2 (1.5%) by GP/community services providers. The remaining 9 (6.5%) were acute-provider services, 6 (66.7%) within neurology services, and 3 (33.3%) where medical input was primarily from psychiatry.

**Table:** 

Acute trust service memory clinics are led by physicians/neurologists, within organisations that primarily focus on hospital or acute care. Community/mental health service memory services are led by psychiatrists, or occasionally primary care doctors, within non-hospital based or mental health service providers.	

Table 1 compares referral rates and staffing between community and acute services.

**Table 1:** A comparison of structural characteristics of services by service type

		Mental health/community services	Acute Services	Overall
Rurality (N=139)	Urban (%)	103 (79.2%)	9 (100.0%)	112 (80.6%)
	Rural (%)	27 (20.8%)	0 (0.0%)	27 (19.4%)
Annual referral rate (N=104) Median (IQR)	Past year	1000 (650.0 – 1590.0)	275 (112.5 – 575.0)	960 (600.0- 1588.0)
	Change relative to 5 years ago	+205 (95.5 - 484.5)	+50 (12.8 – 200.0)	+200 (84.8 - 446.3)
	Per FTE staff member	100.8 (56.7 - 132.8)	45.8 (21.1 - 99.5)	96.8 (51.0 - 131.3)
Median (IQR) staff who are: (N=123)	Doctors	1.5 (1.0 - 2.5)	2.5 (0.5 – 4.0)	1.6 (1.0 - 2.6)
	Nurses	5.1 (3.0 - 8.1)	1.5 (0.4 - 4.1)	5.0 (2.6 - 7.9)
	Occupational therapists	1.0 (0.4 - 2.0)	0.0 (0.0 - 0.5)	1.0 (0.0 - 1.8)
	Clinical Psychologists	0.8 (0.3 - 1.5)	0.5 (0.1 - 1.5)	0.8 (0.3 - 1.5)
	Band 4 Support workers	1.1 (0.0 - 3.4)	0.0 (0.0 - 1.0)	1.0 (0.0 - 3.1)
	Other	1.0 (0.0 - 2.2)	0.1 (0.0 - 1.5)	1.0 (1.0 - 2.0)
Median (IQR) proportion of FTE staff who are: (N=123)	Doctors	14.0% (9.0 - 19.0%)	33.0% (23.0 - 43.0%)	14.0% (9.0 - 20.0%)
	Nurses	45.0% (32.0 - 54.0%)	30.0% (13.0 - 52.0%)	44.0% (30.0 - 54.0%)
	Occupational therapists	8.0% (4.0 - 12.0%)	0.0% (0.0 - 6.0%)	7.0% (1.0% - 12.0%)
	Clinical Psychologists	6.0% (2.0 - 10.0%)	1.0% (1.0 - 2.0%)	6.0% (2.0 - 10.0%)
	Band 4 Therapists & Support workers	12.0% (0.0 - 21.0%)	0.0% (0.0 - 12.0%)	12.0% (0.0 - 21.0%)
	Other	10.0% (0.0 - 18.0%)	1.0% (0.0 - 20.0%)	9.0% (0.0 - 18.0%)

Assuming median service referral rates applied to non-responding services, we estimated there were 192,418 referrals to English memory services in the past year, of which 98.7% (*n* = 189,917) were to community, and 1.3% (*n* = 2,501) to acute services. Community services reported high annual referral rates per FTE memory service staff member (median 100.8, interquartile Range [IQR]: 56.7–132.8.7.8). In these services doctors made up 14.0% (9.0–19.0%) of FTE staff, and 30.0% (20.0–50.0%) of clients were seen at home. All acute services were located in urban areas. Compared to community services they reported fewer referrals per FTE staff (45.8, 21.1–99.5), more doctors (33.0%, 23.0–43.0% of FTE) and saw few patients at home (0.0%, 0.0–15.0%).

Most services accepted referrals from people living in residential care (131/139, 94.2%), with symptoms consistent with advanced dementia (127, 91.4%), complex needs (126, 90.6%) or aged under 65 (126, 90.6%). Around two-thirds accepted referrals of people with learning disabilities (97, 69.8%), or those dependent on drugs or alcohol (89, 64.0%).

### Diagnostic processes (Table 2)

Acute services more frequently reported using Magnetic Resonance Imaging (MRI) brain scans in their diagnostic pathway (7/9, 77.8%) compared with community services (30/128, 23.4%) who more frequently used Computed Tomography (CT) scans (94, 73.4%). Neuropsychological testing to support diagnosis was more available in acute services. Two community services reported no predominant imaging, with an equal proportion of referrals receiving either a CT, MRI, Positron Emission Tomography (PET) or Dopamine Transporter (DAT) scan (1, 0.8%) or an MRI or Single-Photon Emission Computed Tomography (SPECT) (1, 0.8%). Respondents in acute services were more likely, relative to community services, to consider biomarkers likely to change diagnostic practice.

**Table 2:** A comparison of the current and anticipated diagnostic processes of memory services across service type. (All averages reported as medians as data not normally distributed)

		Mental health/community services	Acute Trust	Overall
Main types of brain imaging supporting diagnosis (N=137)	MRI	30 (23.4%)	7 (77.8%)	37 (27.0%)
	CT	94 (73.4%)	2 (22.2%)	96 (70.0%)
	No predominant imaging	2 (1.6%)	0 (0.0%)	2 (1.5%)
	No Imaging	2 (1.6%)	0 (0.0%)	2 (1.5%)
Clinicians can view brain scans (N=139)		79 (60.8%)	8 (88.9%)	87 (62.6%)
Interfacing with neuroradiology (N=138)	No direct interface	30 (23.3%)	0 (0.0%)	30 (21.7%)
	Ad-hoc discussions	59 (45.7%)	5 (55.6%)	64 (46.4%)
	Multi-Disciplinary Team meeting	26 (20.2%)	3 (33.3%)	29 (21%)
	Other	14 (10.8%)	1 (11.1%)	15 (10.9%)
Cognitive testing used (N=138)	Use of translated tools or a translator	88 (68.2%)	5 (55.6%)	93 (67.4%)
	Addenbrookes Cognitive Examination (ACE) (22)	125 (96.9%)	7 (77.8%)	132 (95.7%)
	Montreal cognitive examination (MoCA) (23)	39 (30.2%)	6 (66.7%)	45 (32.6%)
	Mini Mental State Examination (MMSE) (24)	38 (29.5%)	6 (66.7%)	44 (31.9%)
	Rowland Universal Dementia Assessment Scale (RUDAS) (25)	59 (45.7%)	0 (0.0%)	59 (42.8%)
Is neuropsychological testing routinely available (N=138)	For all patients where would support diagnosis	101 (78.3%)	8 (88.9%)	109 (79.0%)
	Prioritised for a limited number of patients	25 (19.4%)	1 (11.1%)	26 (18.8%)
	Not available	3 (2.3%)	0 (0.0%)	3 (2.2%)
Where are clients seen for initial assessment Median proportion (IQR) (N=134)	At home	30.0% (20.0 - 50.0%)	0.0% (0.0 - 15.0%)	30.0% (15.0 - 50.0%)
	In the clinic	62.5% (40.0 - 80.0%)	95.0% (85.0 - 100.0%)	70.0% (40.0 - 70.0%)
	Online/telephone	0.0% (0.0 - 0.0%)	0.0% (0.0 - 3.0%)	0.0% (0.0 - 0.0%)
Can request bloods directly (N= 138)		64 (49.6%)	8 (88.9%)	72 (52.2%)
Anticipated influence of biomarkers on diagnostic practice (N=138)	Will not change practice	28 (21.7%)	1 (11.1%)	29 (21.0%)
	Enhance accuracy in certain areas	67 (51.9%)	5 (55.6%)	72 (52.2%)
	Will be part of routine practice	34 (26.4%)	3 (33.3%)	37 (26.8%)

### Post diagnostic support (Table 3)

Two-thirds (94/139, 67.6%) of services provided further support or monitoring for all patients following diagnosis. Most services (105/138, 76.1%) offered therapies/groups to people after a dementia diagnosis. The most common therapy offered by services was CST, services predominantly provided this in house, 23/138 (16.7%) services referred to other providers. Few services offered additional or alternate groups or therapies. Under half of services offered additional support to minority communities, most commonly translators or referral to charity organisations. 131/138 (94.9%) services offered the opportunity for patients to take part in research; 62/138 (44.9%) services reported higher research engagement over the last 5 years than in preceding years, with only seven (5.1%) reporting lower engagement. These figures were similar across acute and community services.

Acute services felt more ready to prescribe dementia DMTs than community services (8/9 [88.9%] vs. 50/120 [41.7%]) but were less likely to offer post-diagnostic psychological therapy routinely (5/9 [55.6%] vs. 100/129 [77.5%]).

**Table 3:** Provision of post diagnostic support across service type

		Community/mental health services	Acute Trust	Overall
Provision of therapies/groups (N=138)	Cognitive Stimulation Therapy	99 (76.7%)	3 (33.3%)	102 (73.9%)
	Maintaining Quality of Life	23 (17.8%)	2 (22.2%)	25 (18.1%)
	Other cognitive focused groups	8 (6.2%)	0 (0.0%)	8 (5.8%)
	Other therapies*	9 (7.0%)	1 (11.1%)	10 (7.3%)
Total number of therapies offered after dementia diagnosis (N=138)	0	29 (22.5%)	4 (44.4%)	33 (23.9%)
	1	66 (51.2%)	4 (44.4%)	69 (50.0%)
	2	25 (19.4%)	1 (11.1%)	27 (19.6%)
	3+	9 (7.0%)	0 (0.0%)	9 (6.5%)
Change in post-diagnostic support over the past 5 years (N=137)	More support	53 (41.4%)	5 (55.6%)	58 (42.3%)
	Less support	22 (17.2%)	0 (0.0%)	22 (16.1%)
	No change	53 (41.4%)	4 (44.4%)	57 (41.6%)
Specific support for minority communities (N=138)		61 (47.3%)	3 (33.3%)	64 (46.4%)

*Other therapies/groups offered: Adjustment to diagnosis, Couples/family/relationship focused, Stress, anxiety or depression focused

### Mild cognitive impairment

The number of Mild Cognitive Impairment (MCI) diagnoses made increased more in community compared with acute services over the past 5 years (62/125 [49.6%] vs. 2/9 [22.2%]). Acute services more frequently restricted MCI diagnosis to those who received formal neuropsychological assessment, compared with community services (3/9 [33.3%] vs. 20/129 [15.5%]). 26/139 (18.7%) services (all community) routinely offered therapies for people with MCI, most usually groups to help maintain and improve cognitive function (16, 11.5%).

### Cognitive concerns

When describing the cognitive symptoms of individuals without a Dementia, MCI or mood disorder diagnosis, most referred only to absence of dementia; others used the terms functional cognitive/neurological disorder (*n* = 4), or subjective cognitive complaint, decline or impairment (*n* = 3) (Table 4). A quarter of services (36/138, 26.1%) reported offering additional support to these individuals. Most only offered onward referrals (*n* = 11) and/or signposting (*n* = 25). Two referred to brain health clinics. Others provided written resources (*n* = 4) or targeted advice in feedback appointments. One community team manager commented that “for many the consultation (including formulation of memory problems and care plan) is therapeutic in itself”. Three services asked GPs to rerefer for repeat assessments, after 1–3 years. Other services offered: a functional pathway involving “assessment with specific senior practitioner to offer assessment and support and possible signposting”; a brain health pathway, and in one case the MCI group was open to these clients.

**Table 4:** Terms used to describe service users who receive no diagnosis of dementia, MCI or a treatable mood disorder

Term	Frequency
No specific term (recorded as no dementia diagnosis)	18
Functional cognitive disorder	3
Z diagnosis	3
Person with feared complaint but no diagnosis has been made	3
Age related memory issues	1
Brain fog	1
Cognitive impairment due to other causes	1
Functional neurological disorder	1
Subjective cognitive complaint	1
Subjective cognitive decline	1
Subjective cognitive impairment	1

### Comparing service characteristics with local diagnostic rates

The ratio FTE memory service staff to the population aged 65 and over varied from one staff member per 3,020 people to one per 9,379 people across NHS regions. Regions with the highest dementia diagnostic rates (67%+), had more memory service staff relative to local aged 65 + population size (Table 5). Local dementia diagnostic rates varied across NHS regions and were highest in more urban areas. This pattern was reflected in our adjusted multiple linear regression model (R²= 0.25), with local diagnostic rates as the dependent variable. Both rurality (β = 2.14, [95% CI: 1.32, 2.96]) and NHS region (β = 0.70 [95% CI: 0.08, 1.32]) were significant predictors of diagnostic rates. Within our model, no other factors included were significantly associated with diagnostic rates (Table 6).

**Table 5:** Referral and diagnostic rates and FTE staff across NHS region

NHS Region	Median (IQR) referral/FTE rate	Population aged 65+(Pop)	Total FTE in region services	Pop/FTE	Diagnostic rate
Midlands	79.4 (62.3 – 95.9)	2,110,274	225.0	9,379	65.0%
East of England	107.4 (65.1 – 132.5)	1,273,300	204.0	6,241	63.8%
South West	108.6 (97.4 – 143.4)	1,302,863	238.8	5,456	61.2%
South East	78.5 (41.0 – 141.4)	1,847,827	375.0	4,928	63.0%
London	109.4 (71.5 – 150.0)	1,072,807	313.5	3,422	67.4%
North West	87.8 (35.9 – 150.5)	1,413,029	440.8	3,206	70.3%
North East & Yorkshire	74.3 (30.6 – 104.8)	1,619,300	536.6	3,018	67.8%

**Table 6:** Linear regression demonstrating associations between local area diagnostic rates and service characteristics

		Coefficient (β)	Standard Error	t	95% Confidence interval	p-value
Model 1	Referral rate: staff ratio	−0.02	0.01	−2.06	[−0.43, 0.00]	0.042
Model 2	Referral rate: staff ratio	−0.14	0.01	−1.42	[−0.30, 0.00]	0.158
	NHS Region	0.68	0.31	2.21	[0.07, 1.28]	0.029
	Rurality	1.84	0.38	4.89	[1.09, 2.59]	<0.001
Model 3	Referral rate: staff ratio	−0.02	0.01	−1.56	[−0.04, 0.01]	0.130
	NHS Region	0.70	0.31	2.23	[0.08, 1.32]	0.028
	Rurality	2.14	0.41	5.17	[1.32, 2.96]	<0.001
	MSNAP accreditation	−2.18	1.36	−1.60	[−4.88, 0.53]	0.114
	Service type	−2.97	2.71	−1.06	[−8.25, 2.51]	0.292
	Staffing mix (Doctor %)	0.01	0.05	0.17	[−0.09, 0.10]	0.863

### Associations of service characteristics with provision of post-diagnostic therapies (Table 7)

In a fully adjusted logistic regression with receipt of post-diagnostic therapies as dependent variables, only referral rate: staff ratio was an independent predictor of the number of therapies offered (B = 0.01, OR = 1.01, [95% CI: 1.00, 1.02]), indicating that services with higher referral rates were slightly more likely to provide post-diagnostic care.

**Table 7:** Logistic regression demonstrating associations between post-diagnostic therapy provision and service characteristics

		Estimate (B)	Exp(B) (Odds Ratio)	95% Confidence interval	p-value
Model 1	Referral rate: staff ratio	0.01	1.01	[1.00, 1.02]	0.046
Model 2	Referral rate: staff ratio	0.01	1.01	[1.00, 1.02]	0.037
	NHS Region	0.07	1.08	[0.84, 1.39]	0.568
	Rurality	0.13	1.14	[0.85, 1.53]	0.099
Model 3	Referral rate: staff ratio	0.01	1.01	[1.00, 1.02]	0.037
	NHS Region	0.06	1.06	[0.82, 1.37]	0.676
	Rurality	0.17	1.18	[0.85, 1.64]	0.323
	MSNAP accreditation	−0.07	0.93	[0.31, 2.86]	0.905
	Service type	−0.39	0.68	[0.10, 4.69]	0.692
	Staffing mix (Doctor %)	−0.02	0.98	[0.94, 1.02]	0.240

### What changes in the past five years are you most proud of? (Table 1S)

Responses were obtained from 138/139 services; as some individuals responded for multiple services, we received 124 unique responses. We developed five codes describing *preventing well* (providing Brain Health clinics, secondary prevention; testing hearing). 42 codes mapped to *diagnosing well* described improvements in referral triage, training related to diagnosing, a “one stop approach” – where initial assessment and diagnostic feedback were provided in one visit, nurse-led or multiprofessional diagnostic pathways. One service stated they *had “Weekly staff training within Multi-Disciplinary Team (MDT)*,* new staff become confident in diagnosing dementia within months.”* Services also reported increasing diagnostic accuracy – through greater availability of scanning or biomarker testing, or more consistent history-taking and cognitive testing. Four respondents described support for diagnosing in primary care with services stating *“Drive towards supporting primary care diagnosis*,* in turn reducing wait times (as they then don’t need to be referred through the memory service pathway). Facilitated by working together with community dementia nurses who support GPs with doing so. People are able to get timely diagnosis and preserve the memory service for cases that need more specialist contribution.”.* One service reserved assessment slots for people from minority ethnic groups, providing no specific rationale.

81 codes mapped to *supporting well*; 19 to improved integration with other services, at the point of referral (single access point), multidisciplinary meetings, or joint assessments. 18 codes described post-diagnostic group provision including the *“Formalising of post-diagnostic interventions”.* Nine codes describe measures to enhance continuity – through providing a named worker, and/or more capacity for follow-ups, one service described the *“Development of a post-diagnostic service which offers a named worker regular review based on need through a step-up*,* step-down model from diagnosis to end of life.”* Five codes referred to prescribing: non-medical staff involvement in prescribing or post-prescribing monitoring, or readiness for DMTs. Other codes referenced inclusivity of post-diagnostic support, support for people with dementia with specialist needs, or research engagement. 17 codes mapped to *living well*, describing liaison with third sector agencies, outreach and public engagement, this included one service reporting a *“Very close partnership with Age UK who are main providers of dementia advisors”*. 3 codes related to *dying well* (palliative care liaison and Advanced Care Planning).

150 codes related to cross-cutting service work: meeting performance targets, MSNAP accreditation, service redesign, pandemic or post-pandemic work, staffing team attitude or diversity, staff recruitment or retention, changes in staff skill mix, or administrative changes.

### What service changes in the past five years are you most concerned by? (Table 1S)

Responses were reported for 137/139 services, with 119 unique responses. Loss of MCI support was the one code that described preventing well. 33 codes mapped to diagnosing well: describing increased waiting times, and difficulties accessing scanning. One service reported *“Previously no waiting list*,* now up to 160 waiting for diagnosis. Medication is more effective when prescribed earlier. Missing opportunities to assess patients before too advanced.”* Supporting well was described by 21 codes, 10 codes related to post-diagnostic support concerns included *“Lack of post-diagnostic support*,* not just immediate but evidence-based interventions in the community.”* 5 codes mapped to medication including difficulties sending out prescriptions to patients. 11 codes mapped to living well; 4 describing limited carer support and 9 limited community support such as social services such as “*Difficulty in people accessing social services care.”*

### What changes are you anticipating in over the next 5 years? (Table 2S)

Responses were reported for 135/139 services, with 135 unique responses. Nine codes described *preventing well* (linking to community services to address risk factors, providing Brain health clinics, and secondary prevention). 201 codes were mapped to *diagnosing well*. Anticipated changes to pathways to include blood and cerebrospinal fluid (CSF) biomarkers with services reporting the potential of *“Increased use of biomarkers as a factor in decision making.”* Genetic testing and greater neuroimaging access was also key anticipated changes that predominated responses. Services reported on the potential for artificial intelligence to assist in diagnosis, one respondent stated, *“I hope machine learning/AI is used effectively to help in early identification and diagnostic tools such as neuroimaging.”* There was an expectation that more diagnoses would be made in primary care and care homes, and by non-medical prescribers. MDT working and “one-stop shops” were proposed to support earlier, more accurate diagnosis. Notably few respondents discussed the potential impact of more private providers; only two respondents discussed functional assessments. 95 codes were mapped to *supporting well*, with an expectation that DMTs would change diagnostic pathways, with more integrated pathways the most frequently coded responses. Services reported they were *“Hoping to taking on the role of using DMT’s. This would require the service to change how they work.”* Relatively few respondents discussed availability of psychological therapies, or staff training. 23 codes mapped to people *living well*. There was an expectation that third sector organisations would be more frequently commissioned to deliver post-diagnostic support.

## Discussion

This is the largest survey of acute and community memory services in England to date. Findings can inform implementation of treatment advances in England and provide an evidence base of how service structures and processes influence outcomes, for other countries. Further, the format of this survey provides a useful framework for future studies seeking to describe memory service or dementia care provision in other nations.

We found community services see on average twice as many referrals as acute services with half as many doctors, and see more people at home, across urban and rural areas. Two-thirds of services provided further support or monitoring post-diagnosis for people with dementia *and* three-quarters offered post-diagnostic therapies or groups. In line with their workforce predominantly being psychiatrists and mental health nurses, community services were more likely than acute services to provide routine post-diagnostic psychosocial support in the form of therapies or groups. It was surprising that having fewer staff per referral numbers predicted being more likely to offer post-diagnostic support; analyses may not have fully accounted for potential confounders, for example the greater likelihood that community-based, urban services had higher referral rates and greater capability to deliver post-diagnostic groups. Relative to acute services, community services have less access to tests and imaging (MRI scans and directly to blood tests) and perhaps unsurprisingly feel less equipped to implement and deliver DMTs, which will require disease specific biomarkers for entry and MRI scanning both for eligibility and monitoring [26]. Acute services, where neurologists and general nurses are the predominant workforce, felt more prepared for advances in biomarkers and DMTs, though almost half provided no formal psychosocial, post-diagnostic support.

Of almost 200,000 annual referrals to memory services in England, less than 2% are currently made to acute services. The clinical and budgetary implications of shifting even a relatively small, triaged proportion of these referrals and assessments to acute services for the more intensive diagnostic processes acute services provide is substantial. Shifting all would potentially be ruinous. Service modelling, development and change might best focus on upskilling the existing community memory service network to identify patients who would benefit from specialist services provided in acute care, and to develop clearly defined pathways facilitating this. Our community service respondents reflected this view: a quarter felt biomarkers would become part of routine practice in the next five years, and half expected this to enhance diagnostic accuracy for some clients. Strengthening relationships across primary, secondary and tertiary services can also be key for improving dementia diagnosis and care. Integrated Care Systems provide the potential for this [27].

DMTs are expensive now and require intensive monitoring, but costs may change with implementation [8]. Respondents described plans designed to address challenges with volume of referrals and delivery of care such as training other health professionals to diagnose and prescribe, more integrated working in “one stop shops” and collaboration with third sector organisations. Currently only 23% of community services have regular neuroradiology MDT meetings, and only 60% can view scans. Upskilling psychiatrists in community services to interpret brain scans would enhance DMT readiness. Good quality psychological support can save money and increase care quality. There are economic and moral arguments for ensuring NHS commissions evidence-based post-diagnostic psychosocial interventions that improve care, as well as delivering advancement in diagnostics and pharmacological treatment [28].

Acute services were less likely than community services to report increased MCI diagnoses in recent years, restricting the label to those receiving neuropsychological assessment and not providing specific support to this group. Evidence for whether addressing potentially modifiable dementia risk factors in people with memory concerns reduces dementia incidence is limited [29, 30]; some factors, including depression may be part of a prodromal dementia syndrome for some patients [31]. Prevention is prioritised in the Darzi report [5], but there are no NICE guidelines on diagnosing and managing MCI. One in ten services routinely provide support for people with MCI, and a small number for clients who do not receive a diagnosis of dementia, MCI or mood disorder, with variation in terminology and support for this diagnostically heterogenous group. Very few memory services report referring to NHS brain health clinics. Our findings suggest a need for standardisation of care pathways for those with MCI and those with cognitive symptoms without any objective deficits.

We identified marked geographical inequalities: people in regions where memory services were less resourced, and rural areas had less access to timely diagnosis and care. Previous research shows those residing in more deprived areas more commonly receive a ‘unspecified’ dementia diagnosis [32], this work suggests this may be due to variation in memory service resources. The three English NHS regions with highest dementia diagnosis rates had more memory service staff relative to regional 65 + populations. At a service level, main predictors of local dementia diagnostic rates were NHS region and greater rurality.

Our survey has important limitations. There may be responder bias, including towards services under less strain, but it is positive our response rate was high compared with previous research. We did not independently verify data, which may have been subject to desirability bias. Some services may facilitate post-diagnostic support through referral to third sector providers that was not captured. Carer support was not explored in this study due to a focus on primary patient services. Examining the support provided to carers would be a valuable area for future service evaluations, as it plays an important role in overall patient care and wellbeing. Some acute services are tertiary referrals centres, and so their work may not be accurately represented by local diagnostic rates. We mitigated this to some extent through investigating regional relationships between staffing and diagnostic rates.

## Conclusions

Dementia is a complex condition that requires integrated working across medical and social specialties and services, if we are to deliver the inclusive, accurate diagnostic and quality post-diagnostic care that recent advances could enable. Work to map future NHS dementia care pathways is ongoing and should take account of how to ensure existing inequalities, including the stark geographical inequalities identified here, are addressed not worsened.

## Supplementary Information

Supplementary Material 1

## Data Availability

The datasets used and/or analysed during the current study are available from the corresponding author on reasonable request.

## Appendix

### Appendix A: categorization of post-diagnostic interventions

#### Adjustment to diagnosis

Adjusting to Memory Difficulties Group.

Education and Support.

Education for Younger people with dementia.

Post Diagnostic Adjustment Group.

Post Diagnostic Counselling Group/Individual.

Post Diagnostic Information Sessions.

Post Diagnostic Psychological Intervention.

Post Diagnostic Support Group.

Post Diagnostic Therapy.

Training and Education.

#### Stress anxiety or depression

Anxiety Management Group.

Cognitive Behavioural Therapy.

Cognitive Behavioural Therapy for Distressed Carers.

Coping with Forgetting.

Individual Psychology Intervention/Counselling.

Individual Therapy.

Life Story Work.

Lifestyle Matters.

Mood Management.

Worried About Memory Sessions.

#### Help for families and caregivers

Carer Education.

Carer Information.

Carer Support Group.

Carers Day Program.

Carers Group.

Carers Therapy.

Caring and Coping with loss in dementia (For carers).

Creative Writing for Carers.

Information Support Programme.

#### Improving and maintaining cognitive function

Cognitive Stimulation Therapy.

MCI Group.

Memory Group.

Memory Matters.

Memory Strategy Group.

Mindfulness.

#### Maintaining quality of life

Art Group.

Living Well With Dementia.

Music Group/Therapy.

Reading Group.

Recovery College.

Reminiscence Group.

#### Physical health support

Falls Group.

Individual OT programmes.

Occupational Therapy.

Physiotherapy group.

Understanding the Importance of Physical Health.

#### Couples/Families/Relationships/Communication

Dementia Awareness.

Dementia Discovery/Recovery Course.

Dementia Workshops.

Drop in Sessions.

Living at Home with Dementia.

Making Memories Trips.

Memory Support Group.

Men’s Group.

PALS.

Peer Support Group.

Psycho-Ed Group.

Support Groups for Early Stage Dementia.

## Abbreviations

ACE: Addenbrookes cognitive exam
CSF: Cerebrospinal fluid
CST: Cognitive Stimulation Therapy
CT: Computed Tomography
DAT: Dopamine Transporter
DHSC: Department of Health and Social Care
DMT: Disease Modifying Treatment
FTE: Full Time Equivalent
GP: General Practitioner
ICB: Integrated Care Board
LAD: Local Authority District
MCI: Mild Cognitive Impairment
MDT: Multi-Disciplinary Team
MMSE: Mini Mental State exam
MRI: Magnetic Resonance Imaging
MSNAP: Memory Services National Accreditation Programme
NHS: National Health Service
NICE: National Institute for Health and Care Excellence
NIHR: National Institute for Health and Care Research
PET: Positron Emission Tomography
RUDAS: Rowland Universal Dementia Scale
SD: Standard Deviation
SPECT: Single-Photon Emission Computed Tomography
TOP: Total older population
UK: United Kingdom
