# Supplementary material for: A national survey of dementia diagnosis and care in English memory services
Source: BMC Geriatr. 2026 Feb 19;26:406. doi: 10.1186/s12877-026-07155-w (PMC13020397; doi:10.1186/s12877-026-07155-w)
Supplement: Supplementary file 1 — Supplementary Material 1 [file 12877_2026_7155_MOESM1_ESM.docx]

**Supplementary tables**

**Table 1S: Coding framework for responses to question regarding changes in the past five years the service is most proud of, with examples of items coded in each category**

| **Code** | **N** | **Example of items coded** |
| --- | --- | --- |
| **Preventing well (5 codes)** | | |
| Brain health clinic | 3 | *“Brain health clinic - patients are well motivated to try different interventions to change dementia risk factors”* |
| MCI follow-up | 2 | *“MCI list - follow up after 1 year”* |
| Testing hearing | 1 | *“Testing hearing of patients”* |
| **Diagnosing well (42 codes)** | | |
| New referral process | 8 | *“Enhanced triage service for YOD [Young Onset Dementia] patients (having consultant psychiatrist involved at triage stage)”*  *“Screening referrals differently”*  *“Changed triage - accept people who are more appropriate for the service”*  *“Offer waiting well package via the trust recovery college”* |
| Staff training (diagnosis) | 5 | *“Weekly staff training within MDT, new staff become confident in diagnosing dementia within months*  *Regular visiting fellows, teaching how to do assessments and lumbar punctures”*  *“Training of clinicians to deliver diagnosis”* |
| One stop approach | 5 | *“Work to reduce COVID-19 waiting lists with one stop approach”* |
| Biomarker services | 4 | *“Lumbar Puncture very helpful for assessment and diagnosis”*  *“CSF biomarkers - allowed service to be more accurate with diagnosis, treatment can be tailored, increased knowledge about dementias”*  *“Started first CSF biomarker service in a UK mental health trust that is delivered by psychiatrists and mental health nurses”* |
| DIADEM project/ primary care diagnosis | 4 | *“Successful Diadem project to assess service users in care homes”*  *“Drive towards supporting primary care diagnosis, in turn reducing wait times (as they then don't need to be referred through the memory service pathway). Facilitated by working together with community dementia nurses who support GPs with doing so. People are able to get timely diagnosis and preserve the memory service for cases that need more specialist contribution.”* |
| Nurse diagnostic roles | 4 | *“The nurses are doing dementia identification in the care homes and also in their own homes who are very frail, to avoid them coming in to see the doctor”*  *“Nurse led diagnostic clinic”*  *Have trainee advanced practitioners working in this, assisting consultant with diagnostic work* |
| Scanning availability or interpretation | 3 | *“More availability of amyloid PET scans; higher number of metabolic PET scans”*  *“Neurology and neuroradiology MDTs”* |
| Good/ accurate diagnostic assessment | 3 | *“Quality of assessment has increased in terms of accurate diagnosis of dementia sub-types e.g. Lewy-body, Parkinson's, Frontal-temporal lobe Dementia.”* |
| New diagnostic pathways | 3 | *“Multi-professional diagnostic pathway - clinical nurse specialist, specialist OT and consultant psychologist”*  *“Floating consultant clinics”* |
| More consistency | 2 | *“Improved consistency across non-medic clinicians in initial assessments i.e improved clinical history taking quality, improved cognitive screening administration and scoring”* |
| Reducing inequalities | 1 | *“Access for minority ethnic groups - although slots offered, uptake hasn't been huge - suggests more complex factors are involved”* |
| **Supporting well (81 codes)** | | |
| Integrated care | 19 | *“Development and engagement with dementia home treatment team”*  *LAP (local access point): Social care, district nurses, community matrons and mental health service. Service has a worker who sits with LAP everyday and discusses cases to see if specific support is needed. Cluster meetings have built relationships and promoted collaboration”*  *“Wraparound clinic: staff member from social services, admiral nurse, someone from county council and clinician from service working together to provide support after diagnosis, really helpful for patients and their carers alike”*  *“Close links with general hospital and local hospice colleagues… can access EPR, no need to chase wards) - better access to medication records and health updates. Better liaison with ICT (integrated care team) and plans for continuity of care”*  *“Work with primary care network - more contact, smoother process for patients”*  *“Combined psychiatry and neurology clinic - nurses involved in feedback”* |
| Post-diagnostic support | 19 | *“Formalising of post-diagnostic interventions (e.g., CST and LIVDEM)”*  *“Development of groups like CST and recovery through activity”*  *“Improved post diagnostic support, Addition of Living Well groups”*  *“Securing ongoing funding for young onset dementia support group from charity”* |
| Research engagement | 12 | *“Links with research and access to new therapies and support for those living with dementia and carers e.g., START”*  *“People keen to get involved in research”*  *“Maintaining portfolio of research studies”* |
| Continuity of follow-up | 9 | *Development of a post-diagnostic service which offers a named worker regular review based on need through a step-up, step-down model from diagnosis to end of life*  *Discharging of people to GP for monitoring (can be referred back as needed - clearer pathway for this)*  *“Better provisions in place to follow-up with people who are difficult to contact, to check how they are doing”*  *“Dementia advisory service: patients have a named person they can contact”* |
| Prescribing | 6 | *“Introduction of HCAs [Health Care assistants], means they now provide comprehensive post-prescribing monitoring and are trained in phlebotomy and ECGs”*  *“Embracing change - team discussions about new treatments, maintaining awareness of developments”*  *“Development of a multidisciplinary team with a view to DMT's (disease Modifying Treatments)”*  *“We have introduced the non medical prescriber role”*  *“Nurse prescribers able to diagnose, prescribe”*  *“No waiting lists for medication reviews or feedback (thanks to pharmacist joining the team)”* |
| Inclusivity/ reducing inequalities | 6 | *“Access to CST for non-English speakers”*  *“Development/adaptation of Bengali CST”*  *“Offering post diagnostic support to ALL patients”* |
| Carer support/ groups (5) | 5 | *Carer lead in service, carer champions - more support for carers as well*  *Carer group sessions (adapted better now to meet carers other needs and responsibilities)*  *Carers group has been a positive addition (good feedback from carers who say they have gained skills and benefitted from it).*  *Delivery of CRISP (carers education group, Alzheimer's Society type intervention)*  *2 extra admiral nurses working with GP's for the catchment”* |
| Supporting groups with specialist need | 5 | *“Staff with specialist interest areas e.g., young onset, prison referrals, other neurological conditions, learning disabilities to try improve quality of service”*  *“Won award for work with people with dementia who hear voices”*  *“under 65 nurse very good considering complexities of patients”* |
| **Living well (17 codes)** | | |
| Third sector collaboration/ engagement | 9 | *“Close connections with Alzheimer's Society - improved referral rates and support for clients”*  *“Very close partnership with Age UK who are main providers of dementia advisors”*  *“Joint work with xxx Carers and Alzheimer's Society”*  *“Development actions to address inequalities through collaboration with voluntary sector organisations”* |
| Public engagement | 4 | *“Delivering roadshows around memory and services available - very well attended”* |
| Care home liaison/ training | 4 | *“Care Home and reach team set up a training package for dementia - dementia training provided for free to care homes”*  *“Dedicated outreach service in prison service”* |
| ***Dying well (3 codes)*** | | |
| Engaging with palliative care | 2 | *Close links with general hospital and local hospice colleagues*  *Dementia hub - new model developed out of existing resources. Is in collaboration with hospice… Mix of dementia specialist and palliative care specialists* |
| Advanced Care Planning | 1 | *ACPs [Advanced Care Plans] in the memory service* |
| ***Cross-cutting codes (150 codes)*** | | |
| Meeting performance targets | 33 | *“Now in a better position to see people and provide diagnosis within target time periods”* |
| MSNAP accreditation | 22 | *“Working on MSNAP accreditation route for the past 2 years and made improvements”*  *“Achieved MSNAP accreditation, useful as piece of self-reflection”* |
| New clinic structure/ processes | 20 | *“Created new processes and pathways throughout the service in the last 18 months”*  *“Created a standalone memory service”* |
| Pandemic/post-pandemic resilience | 19 | *“Work done to recover from Covid: re-engaging with communities, opening back up local hubs to hold clinics, engagement events with minority communities.”*  *“Flexibility during COVID, offered digital assessments which helped to keep waiting lists down and ensure timely diagnosis for people”* |
| team attitude/diversity | 13 | *“Team reflectiveness, openness to changes, collaborative process within the team”*  *“Very diverse service that reflects the diverse community where the service is located”* |
| staff recruitment/ retention | 11 | *“Recruitment has improved (which was a challenge previously)”* |
| Change in staff skill mix | 8 | *“Changes in skill mix (recruited paramedics, social workers, OTs)”*  *“Now have advanced clinical practitioners in the team”*  *“Development of psychology”* |
| Good patient feedback | 8 | *“Good feedback from patients on the treatments within the service*  *Award for excellent care from service users and family”* |
| Administrative changes | 7 | *“Significant improvements in the administration of clinics - much more streamlined. Centralising booking across 3 separate localities”* |
| Service user engagement | 5 | *“Participation group with carers who contribute to development of CMHT and memory service; part of interview panels, advocate of services”* |
| Staff training (non-specific) | 2 | *“Training up band 6 dementia nurses to be able to provide excellent care in clinic”*  *“Try to do teaching, work with University”* |
| Informational resources | 2 | *“Made YouTube videos which help with every part of process with patients and carers (even before first visit to memory service)”* |

**Table 2S: Coding framework for responses to question regarding changes in the past five years the service is most concerned by, with examples of items coded in each category**

| **Code** | **N** | **Example of items coded** |
| --- | --- | --- |
|  |  | **Preventing well (1 code)** |
| MCI support | 1 | *“Lost MCI pathway groups due to loss of funding, despite it being extremely successful”* |
|  |  | **Diagnosing well (33 codes)** |
| Increased wait for diagnosis | 10 | *“Previously no waiting list, now up to 160 waiting for diagnosis. Medication is more effective when prescribed earlier. Missing opportunities to assess patients before too advanced.”* |
| Increased wait for assessment | 7 | *“Waiting times for initial assessment, 3 month wait at the moment”* |
| Brain scanning access/ report quality | 7 | *“Following Covid, MRI scans provided by acute hospitals, waiting list/times has increased.”*  “Imaging services are also being outsourced, which means the reports coming back aren't good enough and service need to ask for them to be rereported, delaying people's diagnosis and treatment.” |
| Service access through primary care | 3 | *“Clients are having more trouble accessing GP's can also be harder for the service to co-ordinate care with the GP's”* |
| Inappropriate referrals | 2 | *“The increase of referrals from GPs, many of which are not appropriate for memory services, which in turn affects timely provision of services.”* |
| Wait for time for psychology | 2 | *“Longer waiting times for psychology / neuropsychological testing”* |
| Reduced diagnostic quality | 2 | *“During covid continued to complete memory assessments but had to change medium to working by telephone which impacted the quality of cognitive assessments”* |
|  |  | **Supporting well (21 codes)** |
| Post-diagnostic support within service | 10 | *“Lack of provision of post-diagnostic support and lack of focus on this”*  *“Lack of post-diagnostic support, not just immediate but evidence-based interventions in the community”* |
| Medication | 5 | *“Previously, used to work well with pharmacies but in the past few years, it's been difficult to send out prescriptions to people who are unable to come to the service.”* |
| Wait for post-diagnostic support | 4 | *“Increased waiting times, waiting time for post diagnostic support, but new service just been introduced”* |
| Loss of specialism in post-diagnostic care | 1 | *“Post diagnosis is moving to mental health together, concerns that there will be a loss of specialism in older people's dementia care”* |
| DMT delivery uncertainties | 1 | *“Uncertainty around new medications and how that will be delivered”* |
|  |  | **Living well (13 codes)** |
| Limited/variable community (third sector/ social care) support | 9 | *Issues with links with [anon] day services - frequently closing, difficult to make onward referral to these services*  *Difficulty in people accessing social services care (although not directly provided by MS has significant impact on client group)* |
| Carer support/groups | 4 | *“Carers education group stopped”*  *“Lack of evidence-based interventions for carers”* |
|  |  | **Cross-cutting codes (99 codes)** |
| Increased demand, limited capacity | 31 | *“Not enough staff to accommodate the increase in referrals”*  *“As a non-exit service, the demand is out stripping the resource”* |
| General increased wait times | 26 | *“The waiting list - in 2016 the waiting list was 6 weeks. Post COVID it was 9 months, but now 11 months wait”.* |
| Staffing resources | 18 | *“Staffing levels have not increased”* |
| Funding into services | 13 | *“Services like Memory Matters groups, carer support, and admiral nurses, have been cancelled due to funding cuts.”*  *“The lack of ancillary services (e.g., patients are often referred following stroke, and have had no contact with post-stroke rehab services)”* |
| Increasing complexity | 5 | *Complexity of cases (more alcohol dependency, lack to education about alcohol, increased social isolation, more carer stress).* |
| Maintaining/ reporting performance | 3 | “More difficult maintaining KPIs”  *“Major difficulties accessing data about number of assessments and type of diagnosis.”* |
| Change in service pathway | 2 | *Split in the team with the MAS now being a diagnostic service only - it should be one pathway from referral to assessment to post-diagnostic support. Concerned about split, more potential for things to get wrong if patients need to be transferred across services, would be better to have one contained pathway* |
| IT systems | 1 | *Memory service and the GP use System One and consultants, OTs and CPs and OPCMHT use Mobius, same service shouldn't be using two different databases - could do a lot more if one the same database.* |

**Table 3S:** **Coding framework for responses to question regarding anticipated changes in the next 5 years to work with other services, diagnose dementia or anything else, with examples of items coded in each category**

| **Code** | **N** | **Example of items coded** |
| --- | --- | --- |
| **Preventing well (9 codes)** | | |
| Brain health clinics | 3 | *“Developing brain health clinics and looking at prevention, frailty and falls - linking to other aspects of dementia.”* |
| Closer working with community services | 3 | *“Greater integration in the community health spaces and supporting a more preventative model”* |
| More prevention focus | 2 | *“Far greater emphasis on modifiable risk factors and public health approaches”* |
| MCI support | 1 | *“Increased support for patients with MCI (recontact, follow-ups)”* |
| **Diagnosing well (201 codes)** | | |
| New pathways using blood biomarkers | 65 | *“Everybody will have blood biomarkers”*  *“Blood bio-markers can also change our pathway - it could be that GPs can diagnose based on bloods in the future.”*  *“Increased use of biomarkers as a factor in decision making.”*  *“More access to services who provide biomarkers - biomarker testing is getting simpler and cheaper so may be useful in future.”*  *“Routine use of blood biomarkers in people with suspected AD”* |
| New diagnostic pathways | 22 | *“Potentially closer working with acute hospital trusts because of advances in identifying dementia”*  *“Would like to discharge more clients to increase speed of access to assessment and diagnosis.”*  *“Currently streamlining the diagnostic pathway to create capacity in the service.”* |
| Supporting primary care diagnosis/ diagnoses in care homes | 17 | *“Greater emphasis on diagnosing patients in primary care for less complex cases”*  *“Potential opportunity to hand over some of the diagnostic work to GP's.”*  *“doing work with GP practices and care homes to improve relations (improve how service can support GPs to provide the diagnosis)”*  *“More working with GP as working towards GP diagnosing non-complex dementias.”*  *“Greater emphasis on diagnosing patients in primary care, how the service works with primary case and specifically GP's will change”*  *“introducing DIADEM tools in care homes”*  *“Changes in where advanced dementia is diagnosed”* |
| Neuroimaging | 16 | *“Need to have more access to specialist scans like PET”*  *“Will be using AI supported radiology interpretation”*  *“Working with radiology dept to get quantitative volumetric assessments of MRI scans as part of routine practice*  *“I hope machine learning/AI is used effectively to help in early identification and diagnostic tools such as neuroimaging”*  *“Having access to quick neuroimaging would help improve the service. Current delays can increase client anxiety and increases time taken to diagnose”* |
| Implementation of research findings | 10 | *“If new diagnostic interventions/medications are developed in the next 5 years, would like to be involved in that.”*  *“Hoping for new research to improve how dementia is diagnosed, and for early diagnosis”* |
| More non-medical diagnosers | 9 | *“At the moment just doctors who provide diagnosis, got plans in place to extend that ability to other trained professionals (psychologists, non-medical prescribers, ACPs), which should mean seeing more patients and quicker diagnoses”*  *“Nurses taking on more diagnosis after MDT discussions”* |
| Increasing demand | 8 | *“Lower public threshold for seeking a diagnosis once/if the DMT's are licensed (Have already started to see this change)”*  *“Due to the increase in the aging population across the county, referral rates will increase over time.”*  *“More referrals with substance misuse but no one commissioned to assess these people.”* |
| Specialist/multi-professional teams | 8 | *“Increased referrals into specialist hospitals”*  *“Improve MDT working at the point of diagnosis (broaden to have more specialisms involved in discussion).”* |
| Providing earlier diagnosis, including through collaborative approach | 8 | *“Hopefully earlier diagnosis”*  *“If the services was successful in any bids for further funding this would change the service and lead to quicker diagnosis for patients”*  *“Will need to work even more closely with local services, to establish how screening will be done and how patients will be referred back to services” “Better integration and sharing of information through system change, exploring shared clinic space, access to diagnostic testing”* |
| Diagnosis accuracy | 7 | *“We're going to be looking v clearly on our accuracy to ensure those suitable for disease modifying meds get a trial of them - they are not going to suit everyone.”* |
| Genetic testing | 6 | *“Genetic testing - rolling out routine offer of genetic testing for those who are eligible”* |
| One-stop model | 6 | *“Referral to diagnostic time will be quicker as we are introducing 'joint clinics' which are consultant and nurse led. Similar to one-stop-shop model”*  *“A one-stop-shop approach to diagnosis with onsite neuroimaging, cognitive assessment and biomarkers”* |
| CSF biomarkers | 5 | *“Refer patients to lumbar puncture without having to refer them to neurology”*  *“There will be increased use of CSF biomarkers”* |
| Cognitive testing | 5 | *“Increase in use of neuropsychology”*  *“More neuropsychological testing - younger population with potentially more subtle symptoms”*  *“Gamification of cognitive testing”*  *“Looking at cognitive assessment tools for differences in education levels”* |
| Private providers | 3 | *“Other companies might pick up doing memory assessment, not just MAS”*  *“People will start to go private who can afford it and it will become a two-tier system, and then they will go back to NHS services for additional support. Not enough public services resulting huge health inequality where patients from more deprived areas will get less timely diagnoses.”* |
| Virtual appointments | 2 | *“Diagnostics via video conference”* |
| Staff training (diagnosis) | 2 | *“Hoping in next year that staff will be upskilled to be part of diagnostic work (junior staff supervised by consultant)”* |
| Functional (Occupational Therapy) assessments | 2 | *“Look at more functional assessments when more OT staffing is brought in”*  *“Service now has an OT, so functional assessment may be included more thoroughly.”* |
| **Supporting well (95 codes)** | | |
| DMTs will change diagnostic pathway | 54 | *“Hoping to taking on the role of using DMT's. This would require the service to change how they work”*  *“DMTs, if rolled out, will have huge impact on memory service in terms of who prescribes the medication, where that will take place, who provides the scans, etc.”*  *“Faster diagnosis if DMTs involved and reduced waiting times”*  *“If/when new medications become available, will need to identify people earlier and review diagnostic pathway so that more people have access to the medication.”*  *“Advent of DMT's will challenge where priorities lie for NHS care e.g., catching people at earlier stages rather than later stages/more advanced dementia”*  *“New medication might lead to a need for more staff because of increased demand”*  *“More investment in medication”* |
| More integrated pathways | 28 | *“More streamlined referral routes. For secondary mental health and hospital services e.g., Parkinson's”*  *“Integration with health services and social care. Collaborating with range of services as dementia is relevant to all services. Integration with physical health community services would be ideal.”*  *“Integrate more with frailty services”*  *“Moving clinic into premises provided by the acute trust, creating a multidisciplinary team with geriatricians for cerebrovascular and cardiovascular assessment. This will be clinic and research center which will also be able to deliver DMT's”*  *“Closer working with neurology and movement disorders. Hoping to network with neuroradiology.”*  *“With new medication introduced, might need to work more closely with primary care sector”*  *“Care pathways - teams are able to come together like an MDT to look at difficult cases or discuss options for care. Moving away from previous barriers of multiple levels of referrals.”* |
| Psychological therapies and peer support | 7 | *“Large focus on psychological therapies, possibly more working with providers of these therapies”*  “more post-diagnostic support (e.g., would like to start peer support group for living well with dementia)” |
| Staff training | 3 | *“Training/planning for introduction of new drug treatments and how they are administered”* |
| Post-diagnostic support (unspecified) | 3 | *“Development of post-diagnostic service provision in line with new therapy and research findings”* |
| **Living well (23 codes)** | | |
| Third sector collaboration/ engagement | 14 | *“Probably going to see an increased use of third sector organisations to deliver and run groups.”*  *“More work with third sector services but also making them a part of the clinic process.”* |
| Recognising comorbid conditions | 3 | *“identifying depression in older adults earlier”*  *“recognition of co-morbidities”*  *“Looking at more alcohol screening”* |
| Continuity of care | 3 | *“Will provide longer term follow up and have a similar model.”* |
| Engagement with stakeholders | 2 | *“Continued improvement in regular liaison with stakeholders and external services”*  *“Increase in working with experts by experience”* |
| Increasing demands | 1 | *“The service will also see due to the cost of living patients working longer past the usual retirement age, they will need support for employment.”* |
| ***Cross-cutting codes (78 codes)*** | | |
| Concerns that scarcity of resources will affect services | 23 | *“Concern by growing mismatch between what is technically possible and what is actually funded”*  *“The service without proportionate investment may find difficulties in providing what the population needs for health and wellbeing including cognition and further concerns.”*  *“Reduction of available services”*  *“Issues with social care could impact the service”*  *“Ongoing challenges with recruitment of staff may lead to more challenges.”* |
| Increased networking/collaboration | 19 | *“More collaborative work with other services (strengthen what we have now and include those who aren't currently involved, to include a 'one' team format). Sharing of duties/work, but not sure how that will look.”*  *“Engagement with other services will improve (community transformation and involvement with primary care)”*  *“Widening access and collaboration with voluntary health and social services”*  *“More joint working with other services, part of community transformation.”* |
| More inclusive services | 11 | *“We have been considering the establishment of more locally based satellite clinics to try and address issues with rates of access.”*  *“In the next 5 years, hopefully the services raises the profile of young onset dementia and gives it the parity to other dementia diagnoses.”*  *“More work related to BAME community and increasing uptake (engagement work, developing relations so that people are able to come in to seek support)”* |
| Collaboration/Integration | 6 | *“Forsee greater collaboration between services to utilise resources more effectively”*  *“concern over turfing between services as short term and counterproductive attempt to manage excess referrals”* |
| Stronger links with primary care | 6 | *“Strengthening links with primary care and also within the ICB”*  *“Improved integration particularly around primary care”* |
| Structural service changes | 5 | *“Become a commissioned service (not just commissioned for mental health, but separately to develop the service).”* |
| Integration of services (non-specific) | 5 | *“Increased integration between tertiary centers and community services”*  *“Greater integration with other services as part of community transformation”*  *“Service is moving to an OA integrated mental health service.”* |
| Research engagement | 3 | *“Continued focus on dementia e.g., research” “Collaborating with research studies”*  *“more treatment trials of other drugs”* |

**S4: Full survey inclusive of all questions asked to participants**

1. Do you have a service dedicated to memory/dementia assessment?

Yes (Proceed to question 2)

No (answer question 1b & 1c)

(b) What other services does the team delivering dementia diagnosis provide?

_________________________________________________________________________________________________________________________________________________________________________________________________________________________________

(c) What proportion of work is dedicated to:

1. Memory/dementia assessment & diagnosis

________

1. Post-diagnosis services

________

1. What is your staffing mix?

____ FTE Doctors

____ FTE Nurses

____ FTE Occupational Therapists (OT)

____ FTE Clinical psychologists

____ FTE Support Workers (SW)

____ Band 4 therapists

____ Other

If other, please specify:

1. What organisation provides the memory service?

Mental health trust

Acute trust

Community services provider

Health board

GP

Learning disability service

Other

1. Are you a tertiary referral service, for example for people with young onset dementia, rare dementias or learning disability? YES/ NO

Comment:

_________________________________________________________________________________________________________________________________________________________________________________________________________________________________________________________________________________________________________________________________________________________

1. Approximately how many patients were referred to your service in the last year?

____________________

(b) Approximately what was your annual referral rate (number of individuals referred) 5 years ago?

__________________

1. Do you accept referrals from:

|  | People living in residential or nursing homes |
| --- | --- |
|  | People with symptoms consistent with advanced dementia |
|  | People with a learning disability |
|  | People currently dependent on drugs and alcohol |
|  | People with more complex mental health needs (such as schizophrenia) |
|  | People aged under 65 |

(b) For those for whom you do not accept referrals, what advice/alternative pathway do you recommend? [prompt: who do you expect to provide service]

1. Where does your service assess new referrals (Approximate the proportion of initial assessments seen in each setting, for those that do not apply mark as 0%)?

In their homes ___%

In the clinic ___%

Online/Telephone ___%

Other ___%

If other please specify:

1. What cognitive assessment tools do you use in a standard assessment?

Addenbrookes cognitive exam

RUDAS

MMSE

Montreal cognitive examination

Other __________________________________________________________________________________________________________________________________________________

Comment:

_________________________________________________________________________________________________________________________________________________________________________________________________________________________________

(b) What alternative tools do you use (e.g., translated versions of assessment or alternative standardised and validated assessment tools for minority ethic groups)?

_______________________________________________________________________________________________________________________________________________________________________________________________________________________________________________________________________________________________________________________________________________________________________________________

1. What imaging tests do you use routinely in your diagnostic assessment, and what proportion of new referrals receive each modality?

___________________________________________________________________________a._______________________ _______%_________________________________________

b.______________________________%_________________________________________ c.______________________________%__________________________________________ d._______________________________%_________________________________________

1. Do the clinicians in the memory service view scans?

Yes

No

Comment:

_________________________________________________________________________________________________________________________________________________________________________________________________________________________________

1. How do clinicians typically interface with neuroradiology?

Regular MDT meeting

AD-Hoc

No direct interface

Other________________________________________________________________________________________________________________________________________________________________________________________________________________________________________________________________________________________________________

1. Is neuropsychological testing routinely available?

Yes, available for all patients in whom it would support diagnosis

Available for a limited number of clients (for whom it is prioritised)

Not available

1. Can the service request blood tests directly?

Yes

No

Comment:

_________________________________________________________________________________________________________________________________________________________________________________________________________________________________

1. In the next 2 years, how do you think blood biomarker tests will influence clinical decision-making regarding diagnosis?

I don’t anticipate they will change our practice

They will enhance diagnostic accuracy in selected areas

They will be part of routine diagnostic practice

(b) In the event blood biomarkers became available, would your service be in a position to take the bloods?

Yes

No

1. What do you anticipate the role of your service will be in assessing patients for, and prescribing new Disease Modifying Therapies (DMTs)?

Referring to a specialist service

Ordering blood tests/ specialist scans to determine eligibility

Prescribing DMTs

Monitoring progress and side effects

1. How has the proportion of patients diagnosed with MCI changed in your service over the last 5 years?

Increased

Stayed the same

Decreased

(b) If this has changed (Increased/Decreased), what do you think are some of the possible reasons for this change?

_________________________________________________________________________________________________________________________________________________________________________________________________________________________________

1. Is MCI only diagnosed following a formal neuropsychological assessment?

Yes

No

1. After the appointment to feedback diagnosis, does your service provide clients further support or monitoring?

Yes, for all patients

Yes, for patients prescribed medication

Yes, other

No

If other, please specify:

____________________________________________________________________________________________________________________________________________________________________________________________________________________________________________________________________________________________________________

1. What post-diagnostic support is typically provided and for how long?

(a) For patients with lower levels of complexity:

Type of support ________

Number of appointments ___

Duration of appointments ___

Modality _____

Practitioner role _________

Focus on sessions / comments ________________________________________________________________________________________________________________________________________________________________________________________________________________________________________

(b) For patients prescribed anti-dementia medication:

Type of support ________

Number of appointments ___

Duration of appointments ___

Modality _____

Practitioner role _________

Focus on sessions / comments ________________________________________________________________________________________________________________________________________________________________________________________________________________________________________

(b) For patients with higher level of complexity:

Number of appointments ___

Duration of appointments ___

Modality _____

Practitioner role _________

Focus on sessions / comments ________________________________________________________________________________________________________________________________________________________________________________________________________________________________________

1. How has this post-diagnostic support changed in the past 5 years?

Less support

No change

More support

Unsure

Comment:

_______________________________________________________________________________________________________________________________________________________________________________________________________________________________________________________________________________________________________________________________________________________________________________________

1. Do you provide cognitive stimulation therapy (CST)?

Yes, this is provided in-house

Yes, we refer to another service providing this

Yes, this is initially provided in house with maintenance CST provided by another service

We aren’t able to refer but patients can access it locally by self-referral to the provider

No, this is not available

(b) How is this provided?

In person

Online

Both options available

Not sure

1. Do you provide other manualised therapies or groups for people with dementia? [please name them]

Name of therapy _____________________

Group/ individual: __________

Led by (role/ number of facilitators): _____________

Typical number/ frequency/ duration of sessions: ____

Offered to (inclusion criteria): ____________

Approx % clients who receive it: _________________

Focus: ________

Was it: developed in house/ based on a standardised therapy: _____________

Is it conducted to a manual: _____________

1. Do you offer any specific support or services for people from minority communities?

Yes

No

(b) If yes, please tell us about the support do you provide:

__________________________________________________________________________________________________________________________________________________________________________________________________________________________________________________________________________________________________________________________________________________________________________________________________________________________________________________________________

1. Are your patients routinely offered opportunities to take part in dementia research?

Yes

No

1. How has engagement of your clients/service with research changed in the last 5 years?

No change

Lower engagement

Higher engagement

Unsure

(b) What do you think has influenced this?

_____________________________________________________________________________________________________________________________________________________________________________________________________________________________________________

1. Does your service offer any additional support for people with Mild Cognitive Impairment?

Yes

No

Yes/ NO – If YES:

Name of therapy: _____________________

Group/ individual: ______________

Led by (role/ number of facilitators): _____________

Typical number/ frequency/ duration of sessions: ____

Offered to: (inclusion criteria): _________

Approx % clients who receive it: __________

Focus: ___________

Was it: developed in house/ based on a standardised therapy: ____________

Is it conducted to a manual: _______________

26b. Do you provide other support to people with MCI:

Yes

No

If yes, please specify:

____________________________________________________________________________________________________________________________________________________________________________________________________________________________________________________________________________________________________________________________

1. Does your service offer any additional support for people who have cognitive concerns but where assessments do not suggest dementia, MCI or a treatable mood or other major psychiatric disorder?

Yes

No

27b. What diagnostic terms are commonly used by clinicians in your service to describe patients with subjective cognitive complaints that aren’t due to dementia, MCI or a major psychiatric disorder?

_____________________________________________________________________________________________________________________________________________________________________________________________________________________________________________

1. What change in your service in the past 5 years are you most proud of?

__________________________________________________________________________________________________________________________________________________________________________________________________________________________________________________________________________________________________________________________________________________________________________________________________________________________________________________________________________________________

1. What change in the service clients receive in the past 5 years in your service concerns you most?

__________________________________________________________________________________________________________________________________________________________________________________________________________________________________________________________________________________________________________________________________________________________________________________________________________________________________________________________________________________________

1. In the last 5 years, how would you describe any change to:

The accuracy of diagnoses you provide

No change

Lower quality

Higher quality

Unsure

Comments:

______________________________________________________________________________________________________________________________________________________________________________________________________________________________________________________

The quality of care you provide:

No change

Lower quality

Higher quality

Unsure

Comments:

______________________________________________________________________________________________________________________________________________________________________________________________________________________________________________________

1. What changes do you anticipate in your service in the next 5 years:
2. To how you work with other services?

________________________________________________________________________________________________________________________________________________________________________________________________________________________________________________________________________________________________

1. To how you diagnose dementia?

________________________________________________________________________________________________________________________________________________________________________________________________________________________________________________________________________________________________

1. Anything else?

________________________________________________________________________________________________________________________________________________________________________________________________________________________________________________________________________________________________
